# Tofacitinib, an oral Janus kinase inhibitor, as monotherapy or with background methotrexate, in Japanese patients with rheumatoid arthritis: an open-label, long-term extension study

**DOI:** 10.1186/s13075-016-0932-2

**Published:** 2016-01-28

**Authors:** Hisashi Yamanaka, Yoshiya Tanaka, Tsutomu Takeuchi, Naonobu Sugiyama, Hirotoshi Yuasa, Shigeyuki Toyoizumi, Yosuke Morishima, Tomohiro Hirose, Samuel Zwillich

**Affiliations:** Institute of Rheumatology, Tokyo Women’s Medical University, 10-22 Kawada-cho, Shinjuku-ku, Tokyo 162-0054 Japan; The First Department of Internal Medicine, University of Occupational and Environmental Health, Kitakyushu, Japan; Department of Internal Medicine, Keio University, Tokyo, Japan; RA & Inflammation Medical Affairs, Pfizer Japan Inc, 3-22-7 Yoyogi Shibuya-ku, Tokyo, 151-8589 Japan; Pfizer Inc, Groton, Connecticut USA

**Keywords:** Tofacitinib, Japanese, Rheumatoid arthritis, Long-term extension

## Abstract

**Background:**

Tofacitinib is an oral Janus kinase inhibitor for the treatment of rheumatoid arthritis. Here, tofacitinib safety and efficacy data from a long-term extension study in Japanese patients are presented.

**Methods:**

Study A3921041 was a multi-centre, open-label, long-term extension study that included Japanese patients who had participated in a prior Phase 2 or Phase 3 study of tofacitinib as monotherapy or with background methotrexate. Patients received tofacitinib 5 mg twice daily (BID) or tofacitinib 10 mg BID. Dose adjustment of tofacitinib during treatment period, and concomitant usage of disease-modifying antirheumatic drugs including methotrexate after week 12 were permitted. Primary endpoints were adverse events, laboratory parameters and vital signs. Secondary efficacy endpoints included American College of Rheumatology (ACR)20/50/70 response rates, Disease Activity Score (DAS)28-4(erythrocyte sedimentation rate (ESR))<2.6 response rate (DAS-defined remission) and Health Assessment Questionnaire-Disability Index (HAQ-DI) score. Safety and efficacy data were assessed throughout the study.

**Results:**

A total of 486 patients were recruited and treated (1439.9 patient-years of exposure). 308 patients completed the study. Median (range) duration of treatment in this extension study was 1185 (5–2016) days. 476 patients (97.9 %) experienced adverse events; the majority of which (97.8 %) were of mild or moderate severity. The two most common treatment-emergent adverse events were nasopharyngitis (n = 293, 60.3 %) and herpes zoster (n = 94, 19.3 %). For all tofacitinib-treated patients, the incidence rate (patients with events per 100 patient-years) was 10.7 for serious adverse events, 3.3 for serious infections, 7.4 for herpes zoster (serious and non-serious) and 1.2 for malignancies (excluding non-melanoma skin cancer). Mean changes from baseline (start of the index study) in laboratory parameters were consistent with those seen in previously reported studies of tofacitinib. ACR20/50/70 response rates, DAS-defined remission rates and HAQ-DI scores were sustained through to study completion.

**Conclusions:**

Tofacitinib (with or without background methotrexate) demonstrated a stable safety profile and sustained efficacy in Japanese patients with active rheumatoid arthritis. The risk of herpes zoster appears to be higher in Japanese patients treated with tofacitinib than in the global population.

**Trial registration:**

Clinicaltrials.gov NCT00661661. Registered 7 February 2008.

**Electronic supplementary material:**

The online version of this article (doi:10.1186/s13075-016-0932-2) contains supplementary material, which is available to authorized users.

## Background

In Japan current therapies for rheumatoid arthritis include non-biologic and biologic disease-modifying antirheumatic drugs (DMARDs), which can be used alone or in combination with other DMARDs. Methotrexate is the most commonly prescribed DMARD, but its use can be limited by inadequate efficacy, poor tolerability or contraindication. Five tumour necrosis factor inhibitors (infliximab, etanercept, adalimumab, golimumab and certolizumab), an anti-interleukin-6 receptor antibody (tocilizumab) and a selective co-stimulating modulator (abatacept), are currently approved for patients with an inadequate response to traditional DMARDs such as methotrexate [1–3]. Each of these is an injectable, biologic agent. There is currently no oral option available other than tofacitinib for patients in need of therapy after failure of conventional synthetic DMARDs. Therefore, tofacitinib addresses an unmet need for therapeutic options with alternative mechanisms of action, enabling oral formulations.

Tofacitinib is a selective inhibitor of the Janus kinase family and blocks intracellular signalling of multiple key cytokines involved in the inflammatory cascade [4]. Tofacitinib has demonstrated efficacy and safety in the treatment of active rheumatoid arthritis in global phase II, phase III and long-term extension (LTE) studies, and in two randomised, 12-week, phase II studies in Japanese patients with rheumatoid arthritis [5–16].

Double-blind, randomised controlled trials represent the standard approach in determining the short-term safety and efficacy of therapy; however, agents used to treat chronic conditions, such as rheumatoid arthritis, must also demonstrate long-term safety and efficacy. Here we report the safety and efficacy of tofacitinib in Japanese patients in an LTE study, following their participation in phase II and phase III index studies of the drug.

## Methods

### Study design and treatment

Study A3921041 (NCT00661661) was a multi-centre, open-label LTE study conducted at 56 centres in Japan. The study was initiated on 18 April 2008 and included patients who had participated in prior randomised phase II or phase III index studies of tofacitinib in Japan. The primary completion date was 25 December 2013.

The index studies investigated tofacitinib monotherapy 1–15 mg twice daily (BID) (A3921040, NCT00687193) [16] or tofacitinib 1–10 mg BID in combination with background methotrexate (A3921039, NCT00603512; A3921044, NCT00847613) [12, 13]. All patients entering the LTE study received initial treatment with oral tofacitinib 5 mg BID. At the investigator’s discretion, the tofacitinib dose could be increased to 10 mg BID (for inadequate response) or could be reduced from 10–5 mg BID, or it could be temporarily discontinued. Patients who received tofacitinib 10 mg BID for a total of 12 weeks or more are classified herein as the 10 mg BID group, while all other patients are referred to as the 5 mg BID group. The study was not designed for direct statistical comparisons of tofacitinib dosing regimens.

This study was conducted in compliance with the Declaration of Helsinki and the Good Clinical Practice Guidelines established by the International Conference on Harmonisation. The final protocols, amendments and informed consent documentation were reviewed and approved by the Institutional Review Boards and/or Independent Ethics Committee of each study centre (see Acknowledgements for details). All patients provided written, informed consent.

### Patients

Patients were ≥20 years of age (there was no upper age limit) and had completed participation in a prior randomised phase II or phase III index study of tofacitinib for the treatment of rheumatoid arthritis in Japan. Eligibility criteria for the index studies have been reported elsewhere [12, 13, 16]. Briefly, the key inclusion criteria of the index studies were: diagnosis of rheumatoid arthritis based on the American College of Rheumatology (ACR) 1987 revised criteria [17] and active disease (≥6 tender/painful joints (tender joint count (TJC)), 68-joint count) and ≥6 swollen joints (swollen joint count (SJC)), 66-joint count); erythrocyte sedimentation rate (ESR) above the upper limit of normal (local laboratory), or C-reactive protein (CRP) >7 mg/L).

Key exclusion criteria were: severe, progressive or uncontrolled renal, hepatic, haematological, gastrointestinal, metabolic, endocrine, pulmonary, cardiac, neurological, or cerebral disease; current or past history of rheumatic autoimmune diseases other than rheumatoid arthritis (except Sjögren’s syndrome); history of any lymphoproliferative disorder, lymphoma, leukaemia, or lymphatic disease; and evidence of active infection, including untreated or inadequately treated latent tuberculosis. Patients were discontinued from the study if two consecutive clinical laboratory assessments met treatment discontinuation criteria including: two sequential lymphocyte counts <500 cells/mm^3^, two sequential haemoglobin measurements <8.0 g/dL, or a decrease from baseline >30 %.

### Safety assessments

The primary endpoints were adverse event (AE) reports, laboratory safety data and vital signs (irrespective of whether these were reported as an AE). The incidence and severity of all AEs, clinical laboratory tests and vital signs were evaluated throughout the study.

### Efficacy assessments

Efficacy endpoints included ACR20, ACR50 and ACR70 response rates (≥20/50/70 % improvement from baseline, respectively, in both TJC and SJC, as well as in ≥3 of the other five ACR components) [18]. Observed values and changes from baseline in ACR component parameters were assessed: TJC; SJC; Patient’s Assessment of Arthritis Pain (visual analogue scale (VAS) range 0–100 mm); Patient’s Assessment of Disease Activity/Arthritis (PtGA; VAS); Physician’s Assessment of Disease Activity/Arthritis (PGA; VAS); Health Assessment Questionnaire-Disability Index (HAQ-DI; functional impairment in eight categories scored from 0 (no difficulty) to 3 (unable to do) [19]) and CRP.

Other secondary endpoints included HAQ-DI and Disease Activity Score (DAS). DAS was assessed using DAS28-4(ESR) (composite index of four weighted variables: 28 TJC; 28 SJC; ESR; and PtGA), and DAS28-3(CRP), which uses CRP instead of ESR and does not include PtGA [20]. DAS28-4(ESR) and DAS28-3(CRP) remission were defined as DAS28-4(ESR)<2.6 and DAS28-3(CRP)<2.6, respectively [20–22]. Health-related quality of life was assessed using the Short Form-36 (SF-36). The SF-36 health survey (v.2) is a 36-item general health status measure summarised as physical and mental component scores [23].

All efficacy variables were analysed at weeks 2, 4, 8, and 12, and then every 12 weeks (except SF-36, which was assessed every 12 weeks throughout the study).

### Statistical analyses

Baseline values in this study were those of the qualifying index study for all patients. Follow up and AE reporting in this analysis is from the time of enrollment in the LTE study; AEs occurring in the qualifying index studies are not included. The full analysis set included all enrolled patients who had been part of an index study and who received ≥1 dose of open-label study medication in the LTE study; this was equivalent to the safety analysis set. No formal hypothesis tests were conducted; descriptive statistics were calculated for all safety data and efficacy endpoints. Incidence rates (patients with events per 100 patient-years) were calculated as the number of unique patients with an event (for that time period), divided by the total exposure in that treatment group in the pooled cohort, and multiplied by 100. Exposure was censored at the time of the event.

### Protocol amendment

A protocol amendment on 9 March 2009 changed the sample size of the study from 240 to 400 patients to include patients from Study A3921040 [16], extended the study duration, added the option of dose adjustment to 10 mg BID after safety was demonstrated in phase II studies, and removed dose options <5 mg.

## Results

### Patients

**Fig. 1:**
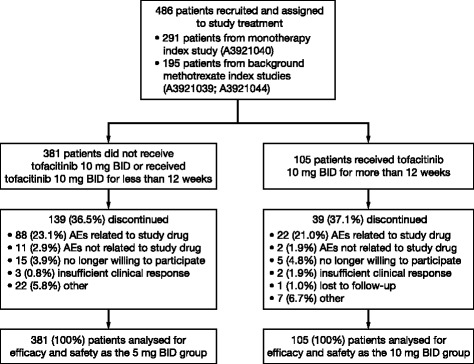
Patient disposition. Evaluation of efficacy was based on patients remaining in the study (observed case analysis); accordingly, not all patients were assessed through to the last observation visit. *AE* adverse event, *BID* twice daily

A total of 486 Japanese patients entered the LTE study: 195 (40.1 %) patients from the tofacitinib with background methotrexate index studies (113 patients from phase II study A3921039 [13] and 82 patients from phase III study A3921044 [12]) and 291 (59.9 %) patients from the tofacitinib monotherapy phase II index study A3921040 [16] (Fig. 1). Of the patients who enrolled in the LTE study from the phase II index studies, 69 came from the placebo treatment arms. All patients from the phase III index study had initiated tofacitinib therapy during the index study. All patients enrolled in the LTE study had initiated tofacitinib therapy within 14 days following the final visit of the index study. All 486 patients received ≥1 dose of study medication and were evaluated for safety and efficacy. At baseline, most patients were female (83.1 %) with a mean (range) duration of rheumatoid arthritis of 7.4 (0.4–45.0) years (Table 1). The median (range) duration of tofacitinib treatment in the LTE study was 1185 (5–2016) days (1439.9 patient-years of exposure). Median (range) duration of treatment for the tofacitinib 5 mg BID group was 1183 (5–2016) days (1111.7 patient-years of exposure), and 1237 (137–1923) days for the tofacitinib 10 mg BID group (328.2 patient-years of exposure). The numbers of patients treated with tofacitinib for ≥1 year, ≥2 years, ≥3 years and ≥4 years (from the beginning of the LTE study) were 423, 321, 278, and 83, respectively.

**Table 1:** Baseline demography and disease characteristics

	Tofacitinib 5 mg BID	Tofacitinib 10 mg BID	Total
	(n = 381)	(n = 105)	(n = 486)
Female, n (%)	318 (83.5)	86 (81.9)	404 (83.1)
Age in years, mean (SD)	53.5 (11.2)	49.3 (11.7)	52.6 (11.4)
Disease duration in years, mean (range)	7.8 (0.4–38.0)	6.1 (0.4–45.0)	7.4 (0.4–45.0)
Tender joint count^a^, n	16.1	17.0	16.3
Swollen joint count^a^, n	13.4	14.3	13.6
HAQ-DI^b^ score	1.23	1.22	1.23
DAS28-4(ESR) score	6.0	6.1	6.0
DAS28-3(CRP) score	5.0	5.2	5.1
ESR, mm/h	50.7	47.7	50.1
CRP, mg/L	24.1	27.4	24.9
Concomitant methotrexate, n (%)	196 (51.4)	26 (24.8)	222 (45.7)
Concomitant systemic corticosteroids, n (%)	256 (67.2)	80 (76.2)	336 (69.1)

Data are mean values except where indicated. Baseline values were those of the index study

^a^Scale 0–68 (tender/painful joints) and 0–66 (swollen joints); higher values indicate greater levels of disease activity

^b^Scale: 0–3; higher values indicate reduced physical function

*BID* twice daily, *CRP* C-reactive protein, *DAS* Disease Activity Score, *ESR* erythrocyte sedimentation rate, *HAQ-DI* Health Assessment Questionnaire-Disability Index, *SD* standard deviation

### Safety

AEs are summarised in Tables 2, 3 and 4. Table 4 includes a summary of tofacitinib exposure and incidence rates for safety events of special interest. The incidences of patients with all-causality and treatment-related AEs were 97.9 % (n = 476) and 96.1 % (n = 467), respectively.

**Table 2:** Summary of safety data up to 288 weeks of observation in 486 patients

Variable	Number (%)
All AEs	476 (97.9)
Serious AEs	139 (28.6)
Discontinuations due to AEs	118 (24.3)
Discontinuations due to serious AEs	75 (15.4)
Dose reduction or temporary discontinuation due to AEs	261 (53.7)
Infections and infestations	
Nasopharyngitis	293 (60.3)
Herpes zoster	94 (19.3)
Bronchitis	51 (10.5)
Upper respiratory tract infection	48 (9.9)
Influenza	48 (9.9)
Cystitis	46 (9.5)
Pharyngitis	46 (9.5)
Gastroenteritis	46 (9.5)
Tinea pedis	34 (7.0)
Oral herpes	33 (6.8)
Injury, poisoning or procedural complications	
Fall	71 (14.6)
Contusion	49 (10.1)
Metabolism and nutrition disorders	
Hyperlipidaemia	56 (11.5)
Vascular disorders	
Hypertension	55 (11.3)
Gastrointestinal disorders	
Dental caries	54 (11.1)
Constipation	43 (8.8)
Diarrhoea	33 (6.8)
Stomatitis	32 (6.6)
Gastritis	26 (5.3)
Nervous system disorders	
Headache	48 (9.9)
Musculoskeletal and connective tissue disorders	
Back pain	46 (9.5)
Investigations	
Lymphocyte count decreased	38 (7.8)
White blood cell count decreased	27 (5.6)
Alanine aminotransferase increased	27 (5.6)
Respiratory, thoracic and mediastinal disorders	
Upper respiratory tract inflammation	34 (7.0)
Cough	33 (6.8)
Skin and subcutaneous tissue disorders	
Eczema	27 (5.6)
Blood and lymphatic system disorders	
Anaemia	26 (5.3)

Treatment-emergent adverse events (*AEs*) affecting ≥5 % of patients in the total population (all causalities) according to Medical Dictionary for Regulatory Activities (*MedDRA*) System Organ Class and MedDRA (v16.1) preferred term. Data are number (%)

**Table 3:** Summary of adverse events and discontinuations over time in the total population

	Month								Post-month 48 (n = 99)
	0–6	6–12	12–18	18–24	24–30	30–36	36–42	42–48	
	(n = 486)	(n = 455)	(n = 430)	(n = 405)	(n = 343)	(n = 309)	(n = 293)	(n = 207)	
Adverse events	379 (78.0)	310 (68.1)	254 (59.1)	215 (53.1)	178 (51.9)	170 (55.0)	145 (49.5)	80 (38.6)	58 (58.6)
Discontinuations due to adverse events	26 (5.3)	19 (4.2)	21 (4.9)	7 (1.7)	13 (3.8)	8 (2.6)	12 (4.1)	1 (0.5)	7 (7.1)
Discontinuations due to serious adverse events	15 (3.1)	16 (3.5)	10 (2.3)	7 (1.7)	6 (1.7)	6 (1.9)	8 (2.7)	1 (0.5)	6 (6.1)
Discontinuations due to serious infection events	6 (1.2)	7 (1.5)	4 (0.9)	4 (1.0)	3 (0.9)	3 (1.0)	5 (1.7)	0 (0.0)	3 (3.0)

Data are number (%)

**Table 4:** Summary of tofacitinib exposure and incidence rates for safety events of special interest

	Tofacitinib	Tofacitinib	All tofacitinib
	5 mg BID	10 mg BID	
	(N = 381)	(N = 105)	(N = 486)
Exposure, patient-years	1111.7	328.2	1439.9
Patients with events per 100 patient-years (95 % CI)			
Adverse events	307.5 (277.0, 340.4)	311.8 (255.0, 377.5)	308.4 (281.3, 337.4)
Serious adverse events	11.2 (9.2, 13.5)	9.2 (6.1, 13.3)	10.7 (9.0, 12.6)
Serious infections	3.2 (2.2, 4.5)	3.7 (1.9, 6.4)	3.3 (2.4, 4.4)
Herpes zoster (serious and non-serious)	7.1 (5.5, 8.9)	8.6 (5.6, 12.7)	7.4 (6.0, 9.1)
Serious herpes zoster	1.0 (0.5, 1.8)	0.9 (0.2, 2.7)	1.0 (0.5, 1.6)
Composite MACE^a^	0.4 (0.1, 1.0)	0.3 (0.0, 1.7)	0.4 (0.1, 0.9)
Gastrointestinal perforations	0 (0.0, 0.3)	0 (0.0, 1.1)	0 (0.0, 0.3)
All malignancy excluding NMSC	1.4 (0.8, 2.3)	0.3 (0.0, 1.7)	1.2 (0.7, 1.9)
Mortality	0.6 (0.3, 1.3)	0.0 (0.0, 1.1)	0.5 (0.2, 1.0)

^a^Total exposure per group is less than for other safety events as composite major adverse cardiovascular event (*MACE*) adjudication applies only to data collected after 25 February 2009. Exposure was 1056.1, 325.0, and 1381.1 patient-years for tofacitinib 5 mg twice daily (*BID*), tofacitinib 10 mg BID and all tofacitinib, respectively

*NMSC* non-melanoma skin cancer

The most common treatment-emergent AEs were nasopharyngitis (60.3 %; n = 293), herpes zoster (19.3 %; n = 94), falls (14.6 %; n = 71), hyperlipidaemia (11.5 %; n = 56) and hypertension (11.3 %; n = 55) (Table 2). Most AEs (97.8 %) were mild or moderate in severity. The overall incidence rate of AEs for all tofacitinib-treated patients was 308.4 patients with events per 100 patient-years (95 % CI 281.3, 337.4; Table 4).

There were 139 patients (28.6 %; 10.7 patients with events per 100 patient-years (95 % CI 9.0, 12.6); Table 4) who had serious AEs (SAEs); 95 (19.5 %) patients had treatment-related SAEs. Most SAEs resolved after tofacitinib discontinuation. The most common AEs leading to temporary discontinuation or dose reduction were nasopharyngitis (14.2 %; n = 69) and herpes zoster (10.9 %; n = 53). For all tofacitinib-treated patients, the overall incidence rate of herpes zoster (serious and non-serious) was 7.4 patients with events per 100 patient-years (95 % CI 6.0, 9.1; Table 4). Herpes zoster was the most common AE leading to permanent discontinuation and this occurred in 12.8 % of all herpes zoster cases (all considered treatment-related). Of the total 94 herpes zoster cases, 14 (1.0 event per 100 patient-years (95 % CI 0.5, 1.6); Table 4) were reported as serious, including one case of disseminated herpes zoster. There were no aural or ophthalmic events. At the end of the study, herpes zoster events had resolved in 88 patients, and were unresolved in 6 patients. Follow up of these patients determined that herpes zoster had resolved or was resolving in four patients and two patients had post-herpetic neuralgia. The investigator judged that there was no need for further follow up to determine stabilised symptoms in these two patients. Thus, herpes zoster events had resolved or were resolving in 92 patients; 2 patients had post-herpetic neuralgia.

The proportion of patients with AEs was highest between 0 and 6 months compared with subsequent 6-month periods (Table 3). Rates of discontinuation due to AEs, SAEs and serious infections also generally decreased over time (Table 3). The overall incidence rate of serious infections was 3.3 patients with events per 100 patient-years (95 % CI 2.4, 4.4; Table 4). Nineteen malignancies (excluding non-melanoma skin cancer) were reported: gastric cancer (n = 3); breast cancer (n = 3); ovarian cancer (n = 2); colon cancer (n = 2); lung cancer (n = 2); and one case each of acute myeloid leukaemia, lymphoproliferative disorder, fallopian tube cancer, thyroid cancer, oesophageal carcinoma, liposarcoma and transitional cell carcinoma. For all tofacitinib-treated patients, the incidence rate of malignancies (excluding non-melanoma skin cancer) was 1.2 patients with events per 100 patient-years (95 % CI 0.7, 1.9; Table 4).

There were seven deaths (1.4 %; all with tofacitinib 5 mg BID; 0.5 patients with events per 100 patient-years (95 % CI 0.2, 1.0); Table 4). Reported causes of death (n = 1 for each) were metastatic ovarian cancer, thrombotic thrombocytopenic purpura, metastatic small-cell lung cancer, gastric adenocarcinoma, rectosigmoid cancer, rheumatoid arthritis associated-pulmonary alveolar haemorrhage-induced acute respiratory distress syndrome, and liposarcoma. All deaths were considered treatment-related, except the case of rheumatoid arthritis associated-pulmonary alveolar haemorrhage-induced acute respiratory distress syndrome. Further details are provided in Additional file 1: Table S1.

**Fig. 2:**
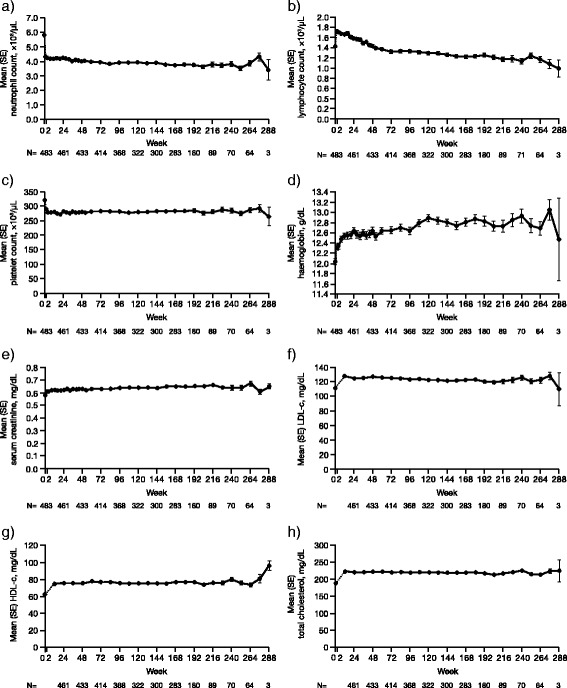
Mean laboratory parameters over time in the total population. Mean neutrophil count (**a**); lymphocyte count (**b**); platelet count (**c**); haemoglobin (**d**); serum creatinine (**e**); low-density lipoprotein cholesterol (*LDL-c*) (**f**); high-density lipoprotein cholesterol (*HDL-c*) (**g**); and total cholesterol (**h**). Baseline values were those of the phase II or phase III index study. *SE* standard error

Changes in means of clinical laboratory parameters were observed, with increases in haemoglobin serum creatine, serum lipids (low-density lipoprotein cholesterol, high-density lipoprotein cholesterol, and total cholesterol), aspartate aminotransferase (AST), alanine aminotransferase (ALT), and total bilirubin, and with decreases in neutrophil, lymphocyte, and platelet counts. With the exception of lymphocyte counts, mean overall changes for clinical laboratory parameters generally occurred within the first month of treatment in the index study and then stabilised with longer treatment duration in this study (Fig. 2).

While mean haemoglobin levels increased with tofacitinib treatment and remained elevated throughout the study, a total of 6 out of 486 patients (1.2 %) had a confirmed (two consecutive measurements) decrease from baseline in haemoglobin that met potentially life-threatening criteria (≥3 g/dL decrease from baseline or haemoglobin ≤7 g/dL; Additional file 2: Figure S1). Of these six patients, one had decreased haemoglobin (below the lower limit of the reference range) at baseline. Onset of decreased haemoglobin levels with life-threatening severity ranged from 77 to 1,099 days following start of tofacitinib treatment in this study. The duration of decreased haemoglobin levels ranged from 6 to 337 days. In all six patients, the decreased haemoglobin either improved or returned to baseline levels, with the discontinuation of the study drug (three patients), or while remaining on the study drug (three patients).

Most instances of neutropenia were mild in severity (mild neutropenia: absolute neutrophil count (ANC) ≥1.5 to <2 × 10^3^/μL); no patients experienced potentially life-threatening neutropenia (ANC <0.5 × 10^3^/μL) (Additional file 2: Figure S1b). Mean increases from baseline lymphocyte counts were seen at week 2, followed by a decline thereafter (Fig. 2b). Elevations ≥3 × upper limit of the normal range in AST, ALT, and total bilirubin were reported in 8 (1.65 %), 20 (4.12 %), and 1 (0.21 %) patients, respectively. No drug-induced liver injury was reported in the study.

### Efficacy

**Fig. 3:**
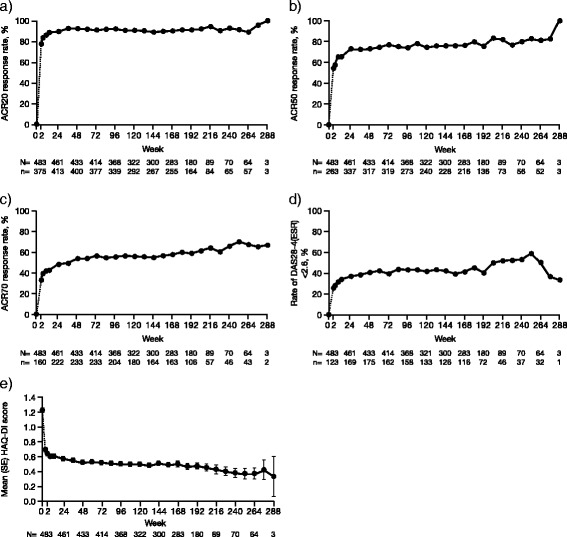
Efficacy endpoints over time in the total population. American College of Rheumatology (ACR)20 **(a)**; ACR50 **(b)**; ACR70; **(c)**; Disease Activity Score in 28 joints (DAS28)-4(erythrocyte sedimentation rate (ESR))<2.6 **(d)**; mean Health Assessment Questionnaire-Disability Index (HAQ-DI) **(e)**. Baseline values were those of the phase II or phase III index study. SE standard error

Evaluation of efficacy over time in this study was based on efficacy responses observed in patients remaining in the study (observed case analysis). The ACR20 response rate at week 12 was 88.6 % and was sustained throughout the study (Fig. 3a). An increase in ACR20 response rate was apparent in patients who, after not responding adequately to tofacitinib 5 mg BID, had their dosage increased to 10 mg BID (Additional file 3: Figure S2a)*.* ACR50 and ACR70 response rates at week 12 were 65.5 % and 42.5 %, respectively, and were also sustained throughout the study (Figs. 3b and c). Changes from baseline in ACR component scores over time are presented in Additional file 4: Figure S3.

The proportion of patients achieving DAS28-4(ESR)-defined remission was 25.5 % (n = 123/483) of patients at week 2 and 49.6 % (n = 69/139) at week 204 (Fig. 3d). The proportion of patients with low disease activity (DAS28-4(ESR)≤3.2 was 42.7 % (n = 206/483) at week 2 and 72.7 % (n = 101/139) at week 204. Mean CRP, DAS28-3(CRP), ESR and DAS28-4(ESR), and rates of DAS<2.6 and DAS≤3.2 over time are presented in Additional file 5: Figure S4. Mean DAS28-4(ESR) for the 5 and 10 mg BID populations over time are presented in Additional file 3: Figure S2b.

A decrease from baseline (−0.63) in mean HAQ-DI score was observed at week 12 that was generally sustained throughout the study (Fig. 3e and Additional file 4: Figure S3f). Mean HAQ-DI scores for both tofacitinib groups over time are presented in Additional file 3: Figure S2c. The proportion of patients with clinically meaningful improvement in HAQ-DI response (≥0.22) was 77.5 % (n = 368/475) at week 12, and was sustained throughout the study (77.7 % (n = 108/139) at week 204).

Improvements from baseline in the SF-36 scores were observed at week 12 and sustained over 120 weeks. The mean (standard error) changes from baseline at week 12 (n = 475) and week 192 (n = 180) were 7.92 (0.34) and 9.29 (0.57), respectively, in SF-36 physical component scores, and 5.06 (0.54) and 2.38 (0.86), respectively, in SF-36 mental component scores.

For all efficacy endpoints, results were similar when patients were stratified by those who had previously received tofacitinib as monotherapy versus those with background methotrexate in their index study (data not shown).

## Discussion

In this LTE study of up to 288 weeks' duration in Japanese patients who had completed a previous phase II or phase III index study, tofacitinib demonstrated a safety and sustained efficacy profile that was generally consistent with the profile seen in the phase II Japanese index studies [13, 16], phase III global studies [5, 9–12, 15] and the global LTE pooled analysis (which included data from the study reported here [14]). Incidence rates of AEs were higher in the current study (308.4 events per 100 patient-years) than in the global pooled analysis (154.5 events per 100 patient-years). Nasopharyngitis was the most common AE observed in both this Japanese study (60.3 %) and the global pooled analysis (12.7 %), and also in the phase II Japanese index studies (12 weeks in duration), although at a lower incidence (8.3 % [13] and 10.2 % [16]) than reported in the present study. The higher overall rate of AEs observed in this study compared with the global LTE pooled analysis is largely due to the higher rates of nasopharyngitis and herpes zoster in this study. In a pooled analysis of long-term tocilizumab therapy in Japanese patients with rheumatoid arthritis, the overall rate of AEs was 465.1 events per 100 patient-years, and the rate of nasopharyngitis was 64.1 events per 100 patient-years [24]. Furthermore, a high rate of nasopharyngitis (68.0 %; 113.0 events per 100 patient-years) was reported in a pooled analysis of etanercept therapy in Japanese patients with rheumatoid arthritis [25]. Given that nasopharyngitis was the most frequently reported AE in the tofacitinib global pooled analysis and that higher rates of nasopharyngitis and overall AEs were also observed in trials of biologic therapies in Japanese patients, reporting bias or local seasonal effects may need to be considered when interpreting the rate of nasopharyngitis (and by extension the overall AE rate) in this LTE study in Japanese patients.

The incidence rate of SAEs observed in this study (10.7 patients with events per 100 patient-years) was comparable to the global LTE pooled analysis (11.1 patients with events per 100 patient-years) [14], lower than that observed in a 5-year extension study of tocilizumab (27.5 events per 100 patient-years) in Japanese patients with rheumatoid arthritis [26], and similar to that reported in the Registry of Japanese Rheumatoid Arthritis Patients for Long-Term Safety (REAL) database, a 3-year study of tumour necrosis factor inhibitors in Japanese patients with rheumatoid arthritis (14.4 events per 100 patient-years) [27].

Incidence rates for serious infections in this study (3.3 events per 100 patient-years) were similar to those reported in the tofacitinib global pooled analysis (3.1 events per 100 patient-years), the Japanese tocilizumab LTE study (5.7 events per 100 patient-years) [26], and to those in the REAL database of Japanese patients treated with tumour necrosis factor inhibitors (5.5 events per 100 patient-years) [27].

In Japan, the incidence of herpes zoster in the general population is estimated at 0.4 cases per 100 patient-years based on a large-scale survey conducted in the Miyazaki Prefecture [28]. In a large observational cohort study, the incidence of herpes zoster in Japanese patients with rheumatoid arthritis was 0.9 cases per 100 patient-years [29]. The relevance of the herpes zoster incidence rate in the Japanese population is highlighted by recent risk-factor analyses that reported an increased incidence rate of herpes zoster in Japanese and Korean patients treated with tofacitinib [30]. It is unclear whether the increased risk was due to genetic, cultural or environmental differences between Eastern and Western populations, and close monitoring of herpes zoster infection rates in Japanese tofacitinib-treated patients is warranted. The incidence rate of all herpes zoster events (serious and non-serious) was higher in the present study (7.1 events per 100 patient-years with 5 mg BID and 8.6 events per 100 patient-years with 10 mg BID) than in the global pooled analysis (4.3 events per 100 patient-years) and also higher than reported in the pooled analyses of tocilizumab therapy (2.3 events per 100 patient-years) [24] and etanercept therapy (2.9 events per 100 patient-years) [25]. The high rate of herpes zoster observed in the current study is consistent with previous risk factor analysis that highlighted high herpes zoster rates in Japanese populations [30]. The incidence rate of malignancies (excluding non-melanoma skin cancer) for tofacitinib-treated patients (1.2 events per 100 patient-years) in this LTE study was comparable with the rate in the global tofacitinib LTE study (1.0 event per 100 patient-years) [14] and with global rates previously reported for biologic DMARDs (0.3–1.77 events per 100 patient-years) [31–34]. Gastric cancer is the most frequently reported malignancy in Japan in the general population [35] and, along with breast cancer, was the most commonly reported type of cancer in the current study. Incidence rates of breast and lung cancer were comparable to those reported in the global LTE study. There was one case of non-melanoma skin cancer (0.07 events per 100 patient-years) reported in this Japanese study compared with 14 cases (0.5 events per 100 patient-years) of non-melanoma skin cancer in the global LTE study.

In this study, deaths occurred in seven patients (1.4 %; 0.5 events per 100 patient-years), with all but one considered treatment-related. The observed rate of mortality was the same as that observed in the tofacitinib global pooled analysis [14], and consistent with that for studies of other DMARDs. The mortality rates in LTE studies of etanercept and adalimumab treatment were 0.8 and 0.7 events per 100 patient-years, respectively [36, 37]. No deaths were reported in the open-label extension phase of the infliximab study [38] and there were no deaths in the tocilizumab study [26], though fewer patients were enrolled in these studies than in the present study and, accordingly, patient exposure was lower.

The proportion of discontinuations from the present study (36.6 %) over 5.5 years was consistent with that reported in the 5-year LTE study of tocilizumab monotherapy in the Japanese population (34.3 %) [26].

With the exception of lymphocyte count, which declined gradually following an initial increase from baseline, changes in clinical laboratory parameters in this study were generally consistent with observations from the index studies [12, 13, 16] and with the global pooled LTE analysis [14], where changes typically occurred during the initial treatment period and then stabilised with longer treatment duration over the course of the LTE study. The proportion of patients who discontinued the study due to abnormalities in clinical laboratory parameters was small. Nevertheless, the contribution of negative selection to the stabilisation of clinical laboratory parameters over time should be considered during the interpretation of these results (i.e., patients who discontinued the study no longer contributed to the mean laboratory parameter values of the remaining total population). Similarly, negative selection may also contribute to the observed decrease in rates of AEs, SAEs and serious infections with time. However, the rate of serious infections was consistent with that reported in Japanese patients with rheumatoid arthritis treated with tocilizumab, where serious infection rates did not increase with long-term tocilizumab exposure [24].

Improvements in all efficacy endpoints evaluated in the current study were generally sustained over 288 weeks and few patients withdrew due to insufficient clinical response. Improvements observed between weeks 2 and 12 may have been due to the dose increases (to 5 mg BID) for many patients who were receiving 1 or 3 mg BID (or placebo) in their previous index study. Similarly, following a lack of efficacy with tofacitinib 5 mg BID, some patients experienced an improvement in clinical response following an increase in dose to 10 mg BID. However, it should be noted that the strength of evidence for a benefit from tofacitinib dose escalation is confounded by the fact that patients could also change their background rheumatoid arthritis medication at any time during the study and by the uncontrolled nature of the observation. Overall, the sustained efficacy data observed in this LTE study suggest that the improvements in signs and symptoms of rheumatoid arthritis and health-related quality of life achieved in the tofacitinib phase II Japanese index studies are maintained in patients who remain on therapy, with nearly 75 % of patients achieving a DAS-defined low disease activity state, and nearly half achieving DAS-defined remission after 4 years.

A key benefit of the study design was that the protocol permitted tofacitinib dose adjustments and the use of concomitant medications, and therefore approximated real-life treatment scenarios. Limitations of this LTE study included the open-label, non-randomised design with no control arm. In the absence of a comparator arm, indirect comparisons of the results in this study were made with external global and Japanese clinical and observational studies. These comparator studies may have different patient populations and study designs; therefore, comparisons should be interpreted accordingly. In addition, it is recognised that LTE studies enroll only those patients who were eligible and completed the preceding randomised clinical trials, a patient population in whom the agent is known to be efficacious and well-tolerated. Although investigators could change the tofacitinib and background therapy dosage to manage the safety and efficacy needs of the patients, this also restricted the ability to compare the different tofacitinib doses. Finally, reduced patient numbers after week 204 limited the data interpretation during the latter stages of the study. The reduced number of patients after this time point reflects the longer time between the beginning of the index study and the end of the LTE study for some patients, rather than a high rate of discontinuation associated with this period of the study.

## Conclusion

In conclusion, tofacitinib 5 mg and 10 mg BID with or without background methotrexate therapy demonstrated a safety profile consistent with that previously reported in the index studies and showed sustained efficacy up to 5.5 years in Japanese patients with active rheumatoid arthritis. The risk of herpes zoster appears to be higher in Japanese patients treated with tofacitinib than in the global population.

## Additional files

Additional file 1: Table S1. Summary of seven patient deaths. (PDF 14 kb) Additional file 2: Figure S1. Patients with decreased haemoglobin and neutropenia over time. (PDF 85 kb) Additional file 3: Figure S2. ACR20 response rate and mean DAS28‑4(ESR) and HAQ-DI over time for patients treated with tofacitinib 5 mg BID and tofacitinib 10 mg BID. (PDF 176 kb) Additional file 4: Figure S3. Mean change from baseline in ACR component parameters over time in the total population. (PDF 72 kb) Additional file 5: Figure S4. Mean DAS parameters and DAS responder rates over time in the total population. (PDF 111 kb)

## Abbreviations

ACR: American College of Rheumatology
AE: adverse event
ANC: absolute neutrophil count
BID: twice daily
CRP: C-reactive protein
DAS: Disease Activity Score
DMARD: disease-modifying antirheumatic drug
ESR: erythrocyte sedimentation rate
HAQ-DI: Health Assessment Questionnaire-Disability Index
LTE: long-term extension
PGA: Physician’s Assessment of Disease Activity/Arthritis
PtGA: Patient’s Assessment of Disease Activity/Arthritis
SAE: serious adverse event
SJC: swollen joint count
TJC: tender joint count
VAS: visual analogue scale
