# Supplementary material for: Tofacitinib, an oral Janus kinase inhibitor, as monotherapy or with background methotrexate, in Japanese patients with rheumatoid arthritis: an open-label, long-term extension study
Source: Arthritis Res Ther. 2016 Jan 28;18:34. doi: 10.1186/s13075-016-0932-2 (PMC4730592; doi:10.1186/s13075-016-0932-2)
Supplement: Additional file 1: Table S1. — Summary of seven patient deaths. (PDF 14 kb) [file 13075_2016_932_MOESM1_ESM.pdf]

Additional table 1. Deaths

1. **Metastatic ovarian cancer.** The patient permanently discontinued from the study on Day 586 due to suspected ovarian cancer, and died on Day 913. The reported cause of death was metastatic ovarian cancer and was considered to be treatment-related. Tofacitinib 5 mg BID was administered for 586 days.
2. **Thrombotic thrombocytopenic purpura leading to multi-organ failure.** The patient permanently discontinued from the study on Day 161 due to cough and fever, and died on Day 230 due to thrombotic thrombocytopenic purpura leading to multi-organ failure. The death was considered to be treatment-related. Tofacitinib 5 mg BID was administered for 161 days.
3. **Metastatic small-cell lung cancer.** The patient permanently discontinued from the study on Day 1099 due to nephrotic syndrome, and died on Day 1313. The reported cause of death was metastatic small-cell lung cancer and was considered to be treatment-related. Tofacitinib 5 mg BID was administered for 1099 days.
4. **Stage-4 gastric adenocarcinoma.** The patient permanently discontinued from the study on Day 200 due to gastric adenocarcinoma, and died on Day 831. The reported cause of death was stage 4 gastric adenocarcinoma with metastases and was considered to be treatment-related. Tofacitinib 5 mg BID was administered for 200 days.
5. **Rectosigmoid cancer.** The patient permanently discontinued from the study on Day 755 due to rectosigmoid cancer, and died on Day 1081. The reported cause of death was rectosigmoid cancer and was considered to be treatment-

	related. Tofacitinib 5 mg BID was administered for 755 days.
**6.**	**Rheumatoid arthritis associated-pulmonary alveolar haemorrhage-induced acute respiratory distress syndrome.** The patient permanently discontinued from the study on Day 1195 due to herpes zoster, and died on Day 1274. The reported cause of death was rheumatoid arthritis associated-pulmonary alveolar haemorrhage-induced acute respiratory distress syndrome and was not considered to be treatment-related. Tofacitinib 5 mg BID was administered for 1195 days.
**7.**	**Liposarcoma.** The patient permanently discontinued from the study on Day 108 for safety reasons, and died on Day 796. The reported cause of death was liposarcoma and was considered to be treatment-related. Tofacitinib 5 mg BID was administered for 108 days.

BID, twice daily
