# Supplementary material for: Tofacitinib, an oral Janus kinase inhibitor, as monotherapy or with background methotrexate, in Japanese patients with rheumatoid arthritis: an open-label, long-term extension study
Source: Arthritis Res Ther. 2016 Jan 28;18:34. doi: 10.1186/s13075-016-0932-2 (PMC4730592; doi:10.1186/s13075-016-0932-2)
Supplement: Additional file 2: Figure S1. — Patients with decreased haemoglobin and neutropenia over time. (PDF 85 kb) [file 13075_2016_932_MOESM2_ESM.pdf]

Additional figure 1. Instances of (a) decreased haemoglobin and (b) neutropenia over time

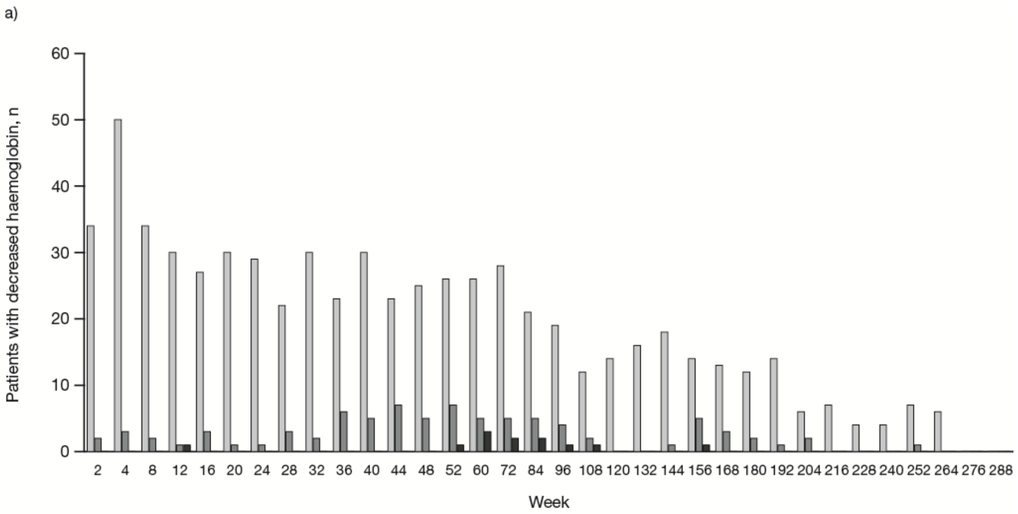

Week	2	4	8	12	16	20	24	28	32	36	40	44	48	52	60	72	84	96	108	120	132	144	156	168	180	192	204	216	228	240	252	264	276	288
Mild to moderate	34	50	34	30	27	30	29	22	30	23	30	23	25	26	26	28	21	19	12	14	16	18	14	13	12	14	6	7	4	4	7	6	0	0
Severe	2	3	2	1	3	1	1	3	2	6	5	7	5	7	5	5	5	4	2	0	0	1	5	3	2	1	2	0	0	0	1	0	0	0
Potential life-threatening	0	0	0	1	0	0	0	0	0	0	0	0	0	1	3	2	2	1	1	0	0	0	1	0	0	0	0	0	0	0	0	0	0	0
N=	483	477	476	475	364	363	461	355	352	441	370	381	433	418	425	414	401	368	343	322	310	300	292	283	237	180	139	89	73	70	70	64	23	3

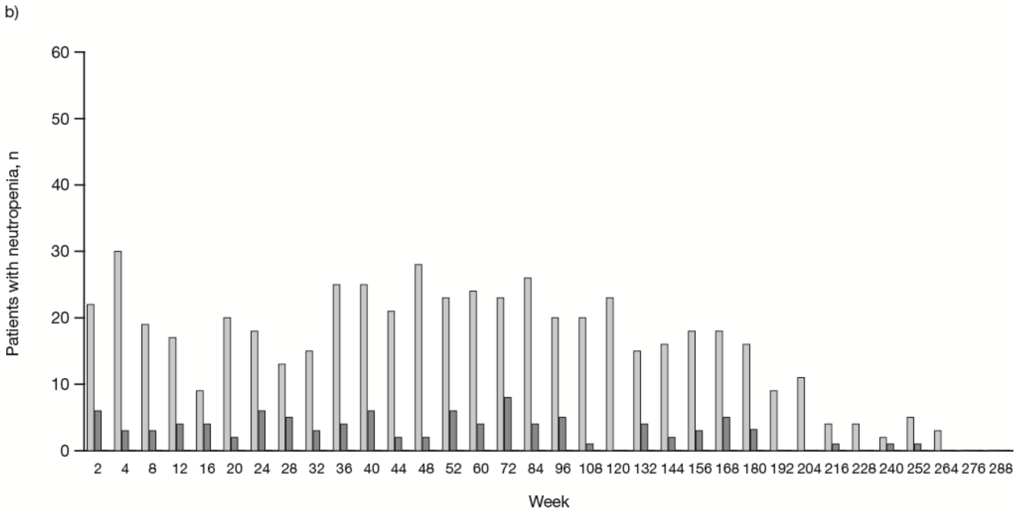

Week	2	4	8	12	16	20	24	28	32	36	40	44	48	52	60	72	84	96	108	120	132	144	156	168	180	192	204	216	228	240	252	264	276	288
Mild	22	30	19	17	9	20	18	13	15	25	25	21	28	23	24	23	26	20	20	23	15	16	18	18	16	9	11	4	4	2	5	3	0	0
Moderate to severe	6	3	3	4	4	2	6	5	3	4	6	2	2	6	4	8	4	5	1	0	4	2	3	5	3	0	0	1	0	1	1	0	0	0
Potential life-threatening	0	0	0	0	0	0	0	0	0	0	0	0	0	0	0	0	0	0	0	0	0	0	0	0	0	0	0	0	0	0	0	0	0	0
N=	483	477	476	475	364	363	461	354	352	441	370	380	433	418	425	414	401	368	343	322	310	300	292	283	237	180	139	89	73	70	70	64	23	3

Decreased haemoglobin: mild to moderate, decrease ≥1 g/dL to ≤2 g/dL; severe, decrease >2g/dL to <3 g/dL or haemoglobin >7 g/dL, but <8 g/dL; potential life-threatening, decrease of ≥3 g/dL or haemoglobin ≤7 g/dL

Neutropenia: mild, 1500-1999 cells/mm^3; moderate to severe, 500-1499 cells/mm^3; potential life-threatening, <500 cells/mm^3
