# Supplementary material for: Tofacitinib, an oral Janus kinase inhibitor, as monotherapy or with background methotrexate, in Japanese patients with rheumatoid arthritis: an open-label, long-term extension study
Source: Arthritis Res Ther. 2016 Jan 28;18:34. doi: 10.1186/s13075-016-0932-2 (PMC4730592; doi:10.1186/s13075-016-0932-2)
Supplement: Additional file 3: Figure S2. — ACR20 response rate and mean DAS28‑4(ESR) and HAQ-DI over time for patients treated with tofacitinib 5 mg BID and tofacitinib 10 mg BID. (PDF 176 kb) [file 13075_2016_932_MOESM3_ESM.pdf]

Additional figure 2. Efficacy endpoints over time in the 5 mg BID and 10 mg BID populations

(a) ACR20; mean (b) DAS28-4(ESR) and (c) HAQ-DI

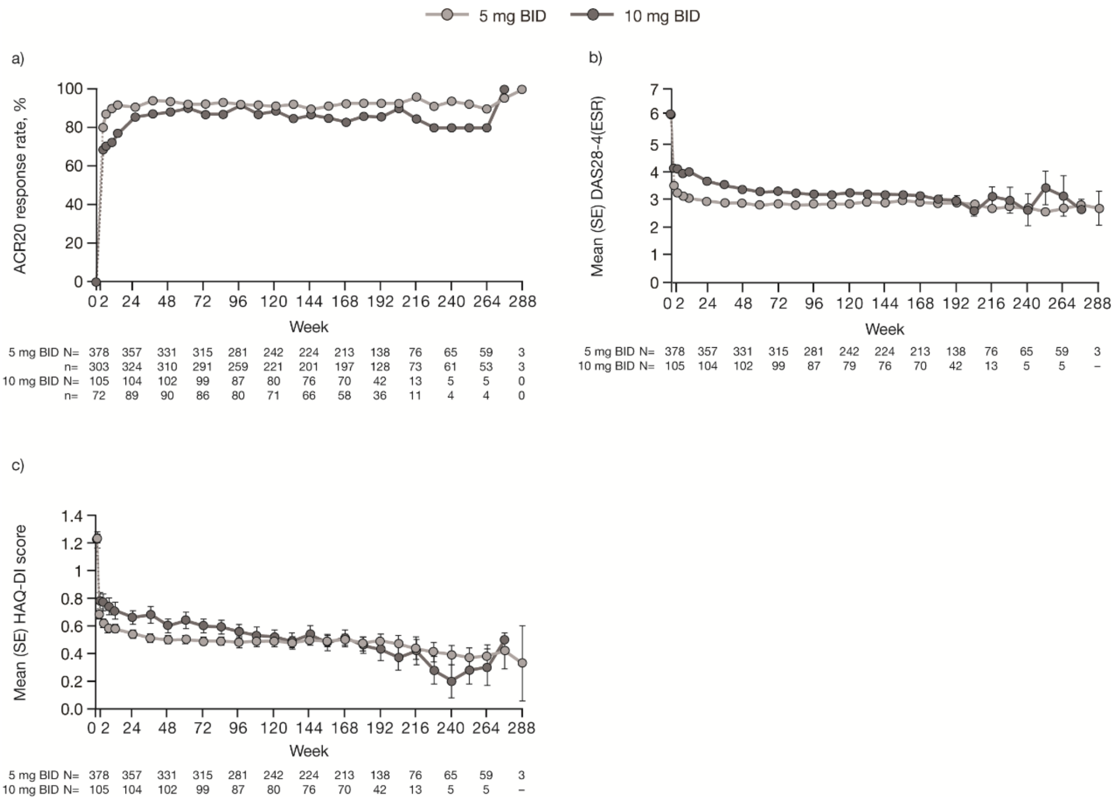

All patients initiated treatment with tofacitinib 5 mg BID. For reasons of inadequate response, tofacitinib doses could be increased from 5 to 10 mg BID. Doses of tofacitinib could subsequently be reduced from 10 to 5 mg BID, or temporarily discontinued. Patients who received tofacitinib 10 mg BID for at least 12 weeks were classed as the 10 mg BID group and all others as the 5 mg BID treatment group.

··· Baseline values were those of the Phase 2 or Phase 3 index study.

ACR, American College of Rheumatology; BID, twice daily; DAS, Disease Activity Score; ESR, erythrocyte sedimentation rate; HAQ-DI, Health Assessment Questionnaire-Disability Index; SE, standard error
