# Supplementary material for: Tofacitinib, an oral Janus kinase inhibitor, as monotherapy or with background methotrexate, in Japanese patients with rheumatoid arthritis: an open-label, long-term extension study
Source: Arthritis Res Ther. 2016 Jan 28;18:34. doi: 10.1186/s13075-016-0932-2 (PMC4730592; doi:10.1186/s13075-016-0932-2)
Supplement: Additional file 4: Figure S3. — Mean change from baseline in ACR component parameters over time in the total population. (PDF 72 kb) [file 13075_2016_932_MOESM4_ESM.pdf]

Additional figure 3. Mean change from baseline in ACR component parameters over time in the total population

Mean change from baseline in (a) TJC; (b) SJC; (c) Pain; (d) PtGA; (e) PGA; (f) HAQ-DI; and (g) CRP

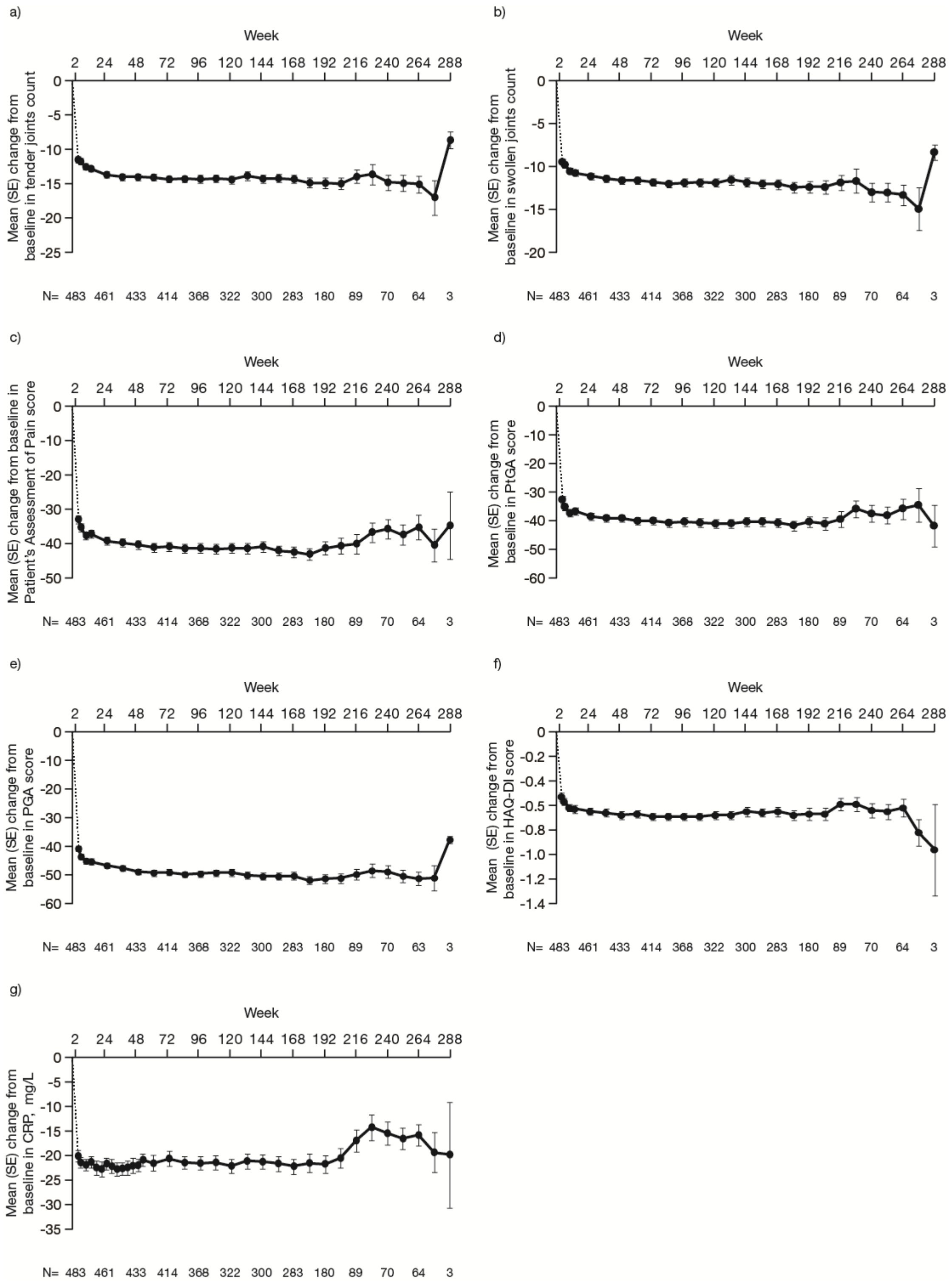

[···] Baseline values were those of the Phase 2 or Phase 3 index study.

CRP, C-reactive protein; HAQ-DI, Health Assessment Questionnaire-Disability Index; PGA, Physician’s Assessment of Disease Activity/Arthritis; PtGA, Patient’s Assessment of Disease Activity/Arthritis; SE, standard error; SJC, swollen joints count; TJC, tender joints count
