# Supplementary material for: Tofacitinib, an oral Janus kinase inhibitor, as monotherapy or with background methotrexate, in Japanese patients with rheumatoid arthritis: an open-label, long-term extension study
Source: Arthritis Res Ther. 2016 Jan 28;18:34. doi: 10.1186/s13075-016-0932-2 (PMC4730592; doi:10.1186/s13075-016-0932-2)
Supplement: Additional file 5: Figure S4. — Mean DAS parameters and DAS responder rates over time in the total population. (PDF 111 kb) [file 13075_2016_932_MOESM5_ESM.pdf]

Additional figure 4. DAS parameters and DAS responder rates over time in the total population

Mean (a) CRP; (b) DAS28-3(CRP); (c) ESR; and (d) DAS28-4(ESR); and rate of (e) DAS28-3(CRP) <2.6; (f) DAS28-3(CRP) ≤3.2; (g) DAS28-4(ESR) <2.6; and (h) DAS28-4(ESR) ≤3.2

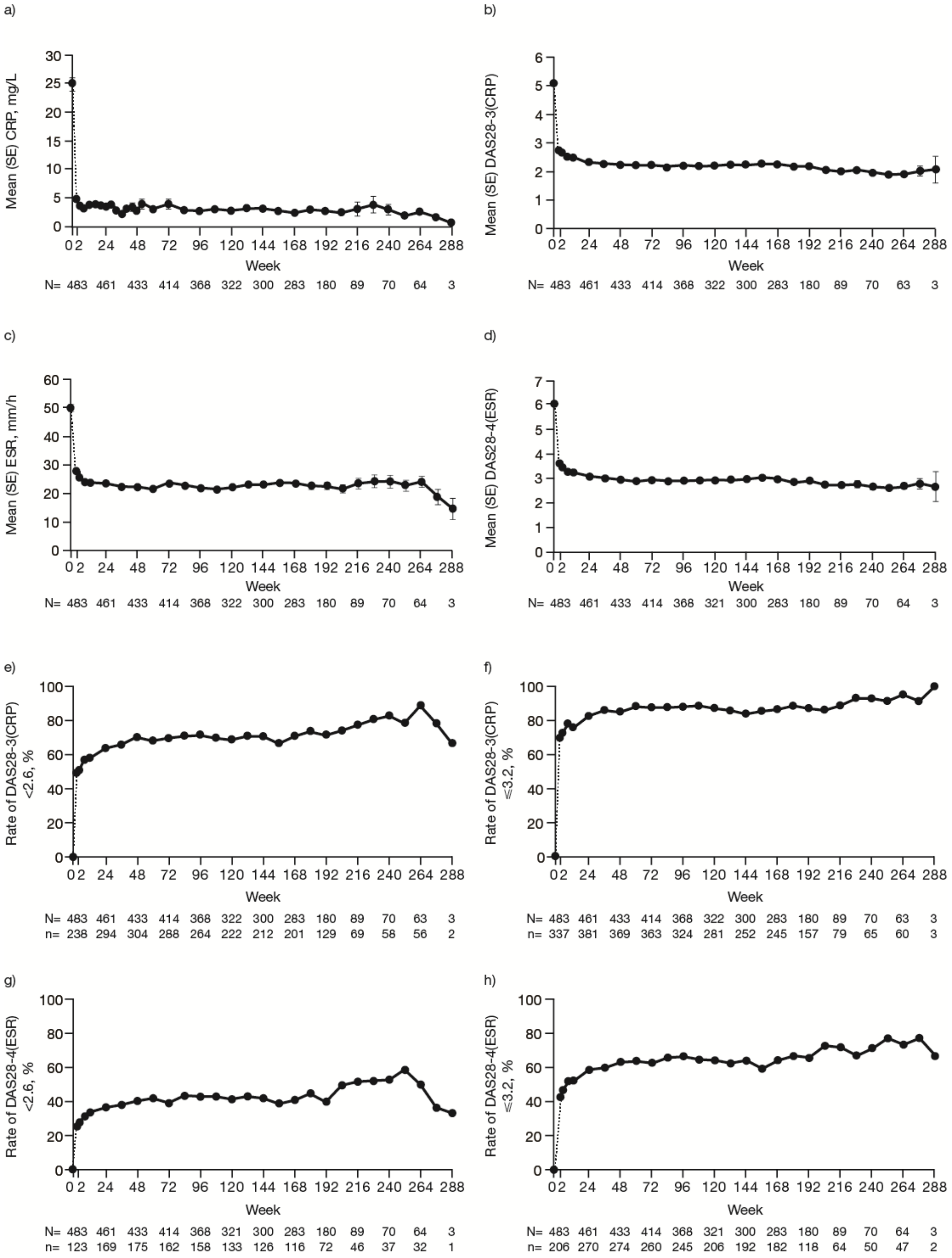

[···] Baseline values were those of the Phase 2 or Phase 3 index study.

CRP, C-reactive protein; DAS, Disease Activity Score; DAS28<2.6: remission; DAS28≤3.2: low level of disease activity; ESR, erythrocyte sedimentation rate; SE, standard error
